# Gitelman syndrome manifesting in early childhood and leading to delayed puberty: a case report

**DOI:** 10.1186/1752-1947-6-331

**Published:** 2012-10-02

**Authors:** Farhan Raza, Mubashar Sultan, Khola Qamar, Ali Jawad, Ali Jawa

**Affiliations:** 1 Temple University Hospital, Philadelphia, PA, 19140, USA; 2 Allama Iqbal, Medical College/Jinnah Hospital Lahore, Lahore, Pakistan

## Abstract

**Introduction:**

Gitelman syndrome is an inherited autosomal recessive renal salt-wasting disorder. It presents with variable clinical symptoms including muscle weakness and fatigue, and the diagnosis is based on metabolic alkalosis, hypokalemia, hypomagnesemia and hypocalciuria. It is usually diagnosed incidentally in early adulthood. There are rare cases of Gitelman syndrome presenting in early childhood; however, to the best of our knowledge it has not previously been associated with delayed puberty.

**Case presentation:**

A 17-year-old South Asian man with recurrent episodes of generalized muscle weakness, fatigue and cramps from the age of two years was admitted for further workup. Before the age of 12 years, the episodes had been mild, but they then got progressively worse. Other symptoms include polyuria, polydipsia, nocturia, paresthesia and occasional watery diarrhea. He also had a history of short stature, poor weight gain and delayed developmental landmarks. His family history was unremarkable except for the consanguineous marriage of his parents. An examination revealed a thin and lean man with blood pressure of 95/60mmHg. His height and weight were below the third percentile and his sexual development was at Tanner Stage II. Laboratory work revealed serum sodium of 124mmol/L, potassium 2.4mmol/L, calcium 6.5mmol/L and magnesium of 1.2mg/dL. His testosterone level was low (0.85ng/mL, normal for his age 2.67 to 10.12ng/mL) with normal levels of luteinizing hormone and follicle-stimulating hormone. The sex hormone findings were attributed to delayed puberty. A 24-hour urinary analysis revealed decreased excretion of calcium (25.9mg/24 hours). Based on the findings of hypokalemic metabolic alkalosis without hypertension, severe hypomagnesemia and hypocalciuria, a diagnosis of Gitelman syndrome was made. Treatment was started with oral supplementation of potassium, magnesium and calcium along with spironolactone and liberal salt intake.

**Conclusion:**

Diagnosis of Gitelman syndrome is usually made incidentally during adolescence or early adulthood based on clinical and biochemical findings. We report that Gitelman syndrome can present during the early childhood years. If undiagnosed and untreated, it can lead to growth retardation and delayed puberty.

## Introduction

Gitelman syndrome (GS) is an inherited autosomal recessive renal disorder [1]. It results from different inactivation mutations in the gene *SLC12A3* on chromosome 16q. This gene encodes the thiazide-sensitive sodium chloride co-transporter channel in the distal convoluted tubule (DCT) of the kidney. Alongside other renal salt-wasting syndromes (Bartter syndrome), GS is considered to be a rare cause of hypokalemic metabolic alkalosis. However, the distinguishing feature of GS is hypomagnesemia and hypocalciuria [2,3]. It presents with variable clinical symptoms including muscle weakness and cramps, fatigue, tetany, vomiting, diarrhea and abdominal pain. It is usually diagnosed incidentally during adolescence or early adulthood. We report a case of GS presenting in early childhood years and the consequences of delayed diagnosis and lack of treatment.

## Case presentation

A 17-year-old South Asian man was referred to our department for recurrent episodes of generalized muscle weakness, fatigue and cramps from the age of two years. Hypokalemia was initially detected at the first episode and he was treated for periodic hypokalemic paralysis. Before the age of 12 years, these episodes were mild, readily improved with ringer lactate and potassium chloride by his treating physician and occurred only twice a year. Beyond the age of 12 years, these episodes gradually worsened in frequency and intensity to the point that they were refractory to previously administered therapies and sometimes required hospitalization.

His symptoms were aggravated by physical activity and hot weather, and were associated with polyuria, polydipsia, nocturia, paresthesias and occasional painless non-bloody diarrhea. He denied the use of any medication, including laxatives and diuretics. He also had a history of short stature and poor weight gain. His birth history was unremarkable but developmental landmarks were delayed. There was no family history of renal salt-wasting syndromes or delayed puberty. However, his parents were first cousins.

An examination revealed a thin and lean, but well-oriented adolescent with no acute distress. His height and weight were below the third percentile (weight 30kg; height 147cm). His blood pressure was 95/60mmHg with orthostatic changes. His sexual development was at Tanner Stage II.

Investigations at the admitting hospital revealed a normal leukocyte count, platelet count, hemoglobin level and erythrocyte sedimentation rate. His urea level was 34mg/dL, creatinine 0.9mg/dL and random blood glucose 105mg/dL. His estimated glomerular filtration rate based on the Modification of Diet in Renal Disease formula was 97.9ml/min per 1.73m^2^. His high blood urea nitrogen level was attributed to mild dehydration due to the hot weather. He was found to have metabolic alkalosis: pH 7.58, HCO_3_^-^ 33.5, base excess +7.6. His serum electrolytes were as follows: sodium 124mmol/L, potassium 2.4mmol/L, calcium 6.5mmol/L and phosphate 3.4mg/dL. His serum magnesium level was not checked at that time. Serum albumin, creatine phosphokinase, free thyroxin, thyroid-stimulating hormone and parathyroid hormone levels were normal.

He was provisionally diagnosed with periodic hypokalemic paralysis and hypocalcemia. Treatment was started with potassium and calcium supplements. After one month, he showed little improvement and he was referred to our department for further evaluation.

Further laboratory tests revealed a low magnesium level (1.2mg/dl) and decreased 24-hour urinary excretion of calcium (25.9mg/24 h). Luteinizing hormone and follicle-stimulating hormone levels were also normal. His level of testosterone was low (0.85ng/mL, normal for his age 2.67 to 10.12ng/mL). Electrocardiography showed a normal sinus rhythm and prolonged QT interval. No abnormality was found on an abdominal X-ray, chest X-ray or brain magnetic resonance imaging. A renal ultrasound revealed a prominent pelvicalyceal system. An insulin tolerance test showed normal cortisol and growth hormone response.

Based on the findings of hypokalemic metabolic alkalosis without hypertension, severe hypomagnesemia and hypocalciuria, a diagnosis of GS was made. No organic cause of hypogonadism was established and the sex hormone findings were attributed to delayed puberty. Treatment was started with oral supplementation of potassium, magnesium and calcium along with spironolactone and liberal salt intake. Our patient and his parents were also counseled about delayed puberty.

A limitation to this case report was that the diagnosis could not be ascertained with sequencing of the implicated gene due to unavailability of genetic sequencing at the presenting hospital (Jinnah Hospital Lahore, Pakistan).

## Discussion

In 1966, Gitelman *et al*. described a familial disorder in three adult female patients with occasional episodes of muscle weakness and tetany. Neither growth retardation nor polyuria was detected. Hypokalemia, hypomagnesemia and hypocalciuria were present [1]. This familial disorder was GS and diagnosis was based on these clinical and biochemical findings.

The prevalence of GS is estimated at approximately 1 in 40,000 and, accordingly, the prevalence of people heterozygous for the condition is approximately 1% in Caucasian populations [2]. However, there is paucity of data about the clinical variability and carrier rate in other populations. GS usually presents after the age of six years and mostly the diagnosis is made only in adulthood [2]. In our patient, the symptoms started at the age of two years in the form of recurrent episodes of muscle weakness. These were mild and were easily treated but gradually became more frequent and increased in intensity, especially after the age of 12 years.

As described before, we postulate that GS can present in early childhood with milder symptoms [3] and become fully symptomatic in adolescence. However, this is the first case to the best of our knowledge in this particular ethnicity.

GS is an autosomal recessive trait. It is caused by missense mutations in the *SLC12A3* gene (located on chromosome 16q) that encodes the thiazide-sensitive sodium chloride co-transporter [2,4]. In *SLC12A3*, 172 distinct mutations have been described, leading to extreme phenotype variability [5]. Female patients with the same mutations are relatively asymptomatic compared with their male counterparts. The nature and position of the *SLC12A3* mutation, combined with male gender, seem to be a determinant factor in the severity of GS [6].

Defects in the thiazide-sensitive sodium chloride co-transporter in the DCT impair sodium and chloride reabsorption in that tubule, causing natriuresis and leading to mild volume contraction. This volume contraction and increased sodium delivery to the macula densa activates the renin-angiotensin-aldosterone axis. Aldosterone increases sodium reabsorption in the cortical collecting duct and leads to increased secretion of potassium and hydrogen ions. This causes hypokalemia and alkalosis [7].

Nijenhuis *et al*. demonstrated hypocalciuria and hypomagnesemia in mouse models for GS and chronic thiazide diuretic use. Hypocalciuria was secondary to enhanced passive calcium reabsorption in the proximal tubule and hypomagnesemia was secondary to the downregulation of magnesium channels (TRPM6) in the DCT [8].

Most patients present with muscle cramps, weakness, paresthesias and episodes of tetany or paralysis. Almost 6% of patients experience hypokalemic paralysis, similar to our patient [7,9]. Other symptoms include nocturia, polydipsia, diarrhea, dizziness and salt craving. These symptoms are attributed to the electrolyte and acid-base abnormalities. The majority of patients also show arterial hypotension. GS can sometimes cause short stature and growth failures [2], but to the best of our knowledge, GS has not previously been reported to cause delayed puberty. We postulate that delayed puberty in our patient was associated with GS, as there was no family history of delayed puberty. The lack of genetic testing prevented us from confirming whether delayed puberty was secondary to the malnutrition or a new pathogenetic etiology.

Hypokalemia and hypomagnesemia lead to a prolonged QT interval in more than 50% of patients with GS. However, cardiac arrhythmias have been described in far fewer patients. Sudden cardiac death has been reported in a few cases [10].

A diagnosis of GS is based on clinical findings and biochemical abnormalities. Typical laboratory findings include hypokalemia, metabolic alkalosis, hypomagnesemia and hypocalciuria. Alongside other renal salt-wasting syndromes (such as Bartter syndrome), GS is considered to be a rare cause of hypokalemic metabolic alkalosis. However, the distinguishing features of GS are hypomagnesemia and hypocalciuria.

Treatment is directed at correcting potassium and magnesium depletion. It requires life-long supplementation and liberal salt intake. Potassium supplementation is with potassium chloride and potassium-sparing diuretics, including amiloride and spironolactone. However, in hypotensive patients, these drugs should be used with caution. Hypomagnesemia is corrected with magnesium chloride (magnesium sulfate or oxide are avoided to prevent diarrhea) [7,11].

## Conclusion

GS can present as early as two years of age. It is an uncommon cause of hypokalemic metabolic alkalosis but, with a typical clinical presentation and biochemical findings, it can be diagnosed. GS has a broad phenotypic variation but few severe forms have been described recently. These severe forms mostly present in male patients and have complications including growth retardation and delayed puberty. It remains unclear if early diagnosis and treatment may prevent these complications.

Antenatal diagnosis of GS is technically feasible but not advised due to the good prognosis in the majority of patients. Given the new findings of severe cases, identifying these patients at an early age may help to prevent complications. Also, the need for and/or benefit of cardiac screening in these patients remain unclear.

## Consent

Written informed consent was obtained from the patient’s father for publication of this case report and any accompanying images. A copy of the written consent is available for review by the Editor-in-Chief of this journal.

## Competing interests

The authors declare that they have no competing interests.

## Authors’ contributions

FR was the main contributor to the preparation of the draft manuscript under the supervision of AJ. MS and KQ contributed to the diagnosis and acquisition of data. AJd critically reviewed the manuscript. All authors read and approved the final manuscript.
